# A Hierarchical Control Strategy for a Rigid–Flexible Coupled Hexapod Bio-Robot

**DOI:** 10.3390/biomimetics8080561

**Published:** 2023-11-21

**Authors:** Kuo Yang, Xinhui Liu, Changyi Liu, Xurui Tan

**Affiliations:** 1 School of Mechanical and Aerospace Engineering, Jilin University, Changchun 130025, China; yagnkuo20@mails.jlu.edu.cn (K.Y.); liuxh@jlu.edu.cn (X.L.); tanxr20@mails.jlu.cn (X.T.); 2 Weihai Institute for Bionics, Jilin University, Weihai 264207, China; 3 Key Laboratory of Bionic Engineering, Jilin University, Ministry of Education, Changchun 130025, China

**Keywords:** multi-legged robot, rigid flexible coupling, hierarchical control, robot dynamics, feedforward compensation, force control

## Abstract

The motion process of legged robots contains not only rigid-body motion but also flexible motion with elastic deformation of the legs, especially for heavy loads. Hence, the characteristics of the flexible components and their interactions with the rigid components need to be considered. In this paper, a hierarchical control strategy for robots with rigid–flexible coupling characteristics is proposed. This strategy involves (1) leg force prediction based on real-time motion trajectories and feedforward compensation for the error caused by flexible components; (2) building upon the centroid dynamics model of the rigid-body chassis, the centroid trajectories (centroid angular momentum (CAM) and centroid linear momentum (CLM)) and the body trajectory are taken into account to derive the optimal drive torque for maintaining body stability; (3) finally, the precise force control of the hydraulic drive units is achieved through the sliding mode control algorithm, integrating the dynamic model of the flexible legs. The proposed methods are validated on a giant hexapod robot weighing 3.5 tons, demonstrating that the introduced approach can reduce the robot’s vibrations.

## 1. Introduction

In recent years, the exploration of increasingly unstructured environments has led to significant advancements in legged robotics [1,2,3,4], particularly large-scale legged robots driven by hydraulic systems, which demonstrate both carrying capacity and operational ability [5,6]. However, the elasticity-induced deformation of the legs in heavy-duty robots during motion [7] impacts the overall stability of the system and the tracking accuracy of the motion trajectory. The coupling of motion between flexible and rigid systems is highly complex, and the flexible deformation of the legs can affect energy consumption and induce body oscillation in the robot [8].

There is extensive current research on rigid–soft coupling robots, with the most direct solution being the optimization of the robot structure based on modern design methods [9,10]. K. Xu [11] proposed an optimized design method for the flexible joint linkage of a quadruped robot, which improves the payload capacity of the robot. However, for existing robots, it is challenging to modify their structures, necessitating research from a control perspective. T. Zhang [12] and Chen, T [13] proposed a pre-adaptive input shaping method to suppress the residual vibration. K. Zheng [14] utilized the singular perturbation principle to decompose the motion into slow and fast subsystems with different time scales. However, the vibration mode of legged robots is forced vibration, and the aforementioned methods do not yield ideal control results for such vibrations.

Xun, M. [15] and Pan, T. [16] studied active control of flexible components by designing a piezoelectric actuator. The issue of rigid–flexible coupling in mechanisms has also been widely studied in aerospace [17]. Zhong, R [18] utilized neural networks to identify rigid–soft coupling terms and improved attitude control precision through an enhanced PD controller. Ye, D [19] proposed a robust output feedback attitude-tracking control method, ensuring that the rigid–flexible spacecraft could track time-varying reference attitudes based solely on angle and angular velocity measurements. The feedback from the actual position information of the legs of a legged robot is difficult to obtain, and its body vibration is primarily caused by the coupling between the rigid body and the flexible legs. Therefore, the methods above do not apply to legged robots.

The error caused by the flexible components feedforward compensation method has proven effective on industrial robotic arms [20]. X. Chen [21] established an error model for robot rigid–soft coupling, improving robotic accuracy through non-kinematic calibration of industrial robots. Deng, K [22] employed an external measurement system and a compliance model to measure or estimate compliance errors, subsequently using control algorithms to compensate for these errors. If flexible deformation can be accurately predicted, it can ensure force stability in the robot’s parallel mechanisms during support, inhibiting the majority of oscillations. The movement of legged robots primarily relies on supporting legs bearing the entire vehicle’s weight; the deformation of the legs is directly influenced by the forces they endure. Therefore, the prerequisite for feedforward compensation is an accurate leg dynamics model. Dynamic modeling is mainly achieved through Euler’s method and Lagrange’s method [23], with common modeling methods for flexible bodies being the finite-element method [24,25] and assumed-modes method [26,27]. Ren [28] derived the rigid–soft coupled dynamic equations for a parallel-legged hexapod robot based on the assumed-modes method and Lagrange equations. Through dynamic analysis, it was demonstrated that the impact of leg elasticity deformation on the motion characteristics of legged robots during movement cannot be ignored.

Research on the control strategies for suppressing oscillations in flexible robots has focused on impedance control based on the position of inner loops and the force of outer loops. References [29,30,31], respectively, utilized the principle of impedance control on bipedal, quadrupedal, and hexapod robots, conducting research on the motion control of flexible robots based on feedback from force sensors set at the foot ends. However, for deformable flexible mechanisms, it is difficult to achieve effective force tracking based on position impedance control, and due to the influence of impedance model parameters, the system may exhibit steady-state errors [11,32]. The deformation of the legs is minimal relative to the robot’s range of motion, but because the supporting process involves a parallel mechanism, the coupling relationship between the body and each leg leads to oscillations in the robot.

**Figure 1:**
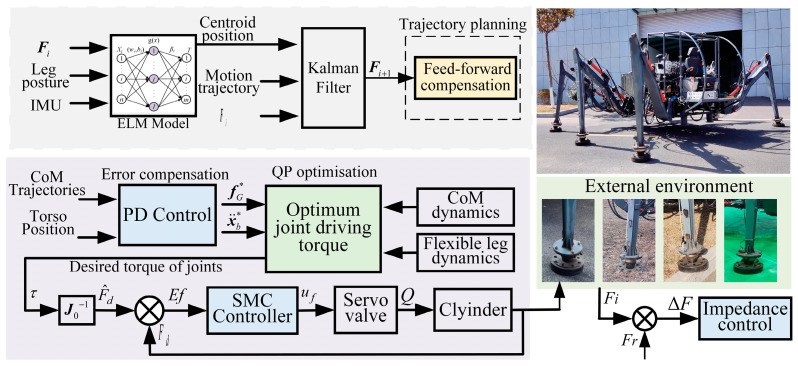
Schematic diagram of hierarchical control strategy.

Therefore, this article proposes a hierarchical control strategy based on the rigid–soft coupling characteristics of the robot, as shown in Figure 1. The robot’s real-time center-of-gravity position is obtained through the robot’s inertial measurement unit, foot-end force sensors, and cylinder position sensors. Then, the real-time foot-end force is predicted using the Kalman algorithm, and feedforward compensation is performed for single-leg flexible deformation, reducing the deviation due to flexible deformation between the base of each leg and the foot-end position. To improve the robot’s terrain adaptability, impedance control based on the position of the inner loop is employed at the initial extremities in contact with the ground. A precise force control method is used when the foot-end force reaches the target threshold. First, the trajectory tracking of the CAM [33], CLM, and the body trajectory were conducted to obtain the desired driving torque of the legs. Then, the sliding mode control algorithm is used to accurately control the output force of the electro-hydraulic servo valve control cylinder system, eliminating the impact of the remaining deviation from feedforward compensation and disturbances on the robot body.

The main contributions of this paper are as follows:

(1) Feedforward compensation for robot flexible deformation is proposed, predicting the center of gravity and foot-end force for feedforward compensation of flexible deformation.
(2) A coordinated control strategy is proposed involving hydraulic drive unit force control and leg impedance control, along with a controller based on the rigid-body trunk and flexible-leg models. The desired drive force/moment is obtained by a quadratic programming algorithm using the CAM, CLM, and torso trajectories as objective functions.

The proposed control method reduces the body vibration caused by flexible deformation.

The remainder of this paper is organized as follows: Section 2 describes the kinematics and dynamics modeling of the legs. Section 3 presents the hierarchical controller. Section 4 discusses the results of experiments and simulations, and the conclusion is presented in Section 5.

## 2. Related Work

**Figure 2:**
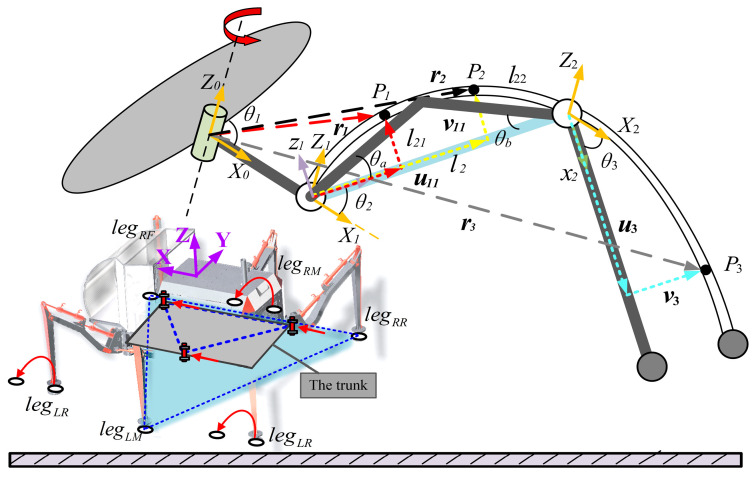
Schematic diagram of the overall structure and the flexible deformation of the legs.

In this paper, we have developed the insect-like hexapod biomimetic robot shown in Figure 2. It is composed of six three-link rigid–flexible mechanical legs. The D-H parameters of the structure of each leg are shown in Table 1.

The motion schematic of the robot is shown in Figure 2. During movement, all the legs of the robot are divided into two parts: one is supporting, and the other is swinging. Legs in the swinging state only need to overcome their own weight. In contrast, legs in the supporting state bear the weight of the entire robot and are subjected to greater forces, producing corresponding deformations. Therefore, this paper mainly studies the supporting legs in the supporting state.

The schematic diagram of the elastic deformation of the leg under the force during the support stage is shown in Figure 2. The linkage mechanism in the middle of the thigh includes the constraint of the hydraulic cylinder hinge point, so the flexible thigh cannot be simplified as an Euler beam. Thus, the thigh is divided into two parts, lengths *l*_21_ and *l*_22_, respectively. The angle between the decomposed thigh and the line connecting the head and tail joints is *θ_a_* and *θ_b_*, respectively, then *l*_2_ = *l*_21_*cosθ_a_*+*l*_22_*cosθ_b_*. The lower leg is a slender rod with small elastic deformation, described by the Euler–Bernoulli beam. When the robot walks, the ratio of leg axial deformation and shear deformation is small, so this paper only analyzes the bending deformation of the leg linkage. The situations of shear and torsion have not been taken into account.

The representation of vector $r_{k}$ at position *P*_1_ after deformation of point *P* in relation to the inertial coordinate system is as follows:

(1) rk=r0+Rk0(uf+vf)

where $r_{0}$ denotes the displacement vector of the floating coordinate system under the inertial coordinate system, Rk0 denotes the rotation matrix from the floating coordinate system to the inertial coordinate system, and $u_{f}$ and $v_{f}$ denote the vector representations of an arbitrary point under the floating coordinate system before deformation and after flexible motion, respectively.

According to the assumed modal method, the deformation $v_{f}$ within the floating coordinate system is described in a discretized manner as:

(2) $$v_{f}=\sum {}_{i=1}^{n}a_{i}(t)\Phi_{i}(x)$$

where $a_{i}(t)$ is the modal coordinate, and $\Phi_{i}(x)$ is the modal function.

The total kinetic energy of a single leg is the sum of the rigid-body base part, the flexible thigh, and the kinetic energy of the flexible calf, where the kinetic energy of the flexible body is expressed as:

(3) $$T=\frac{1}{2}\int_{0}^{L}\rho {\dot{r}}^{T}\dot{r}dl=\frac{1}{2}{\dot{q}}^{T}M\dot{q}$$

where ρ is the material density, *l* is the length of the leg connecting rod, $\dot{q}$ is the generalized velocity coordinate, and ***M*** is the connecting rod mass matrix.

The position of point *P*_1_ on the flexible thigh link *l*_21_, under the *X*_0_*Y*_0_*Z*_0_ coordinate system, is expressed as:

(4) $$r_{1}=A_{1}(u_{11}+v_{11})+l_{1}$$

where ***u***_11_ and ***v***_11_ represent the vectors of *P*_1_ before and after deformation in the floating coordinate system, and *A*_1_ represents the rotation transformation matrix of the thigh floating coordinate system *x*_1_*y*_1_*z*_1_ in *X*_0_*Y*_0_*Z*_0_. The parameters are as follows:

(5) $$\left\{ \begin{array}{c} u_{11}={\left[\begin{array}{ccc} x_{1} & 0 & 0 \end{array}\right]}^{T},v_{11}={\left[\begin{array}{ccc} 0 & 0 & v_{1} \end{array}\right]}^{T},l_{1}={\left[\begin{array}{ccc} l_{1} & 0 & 0 \end{array}\right]}^{T}\\ A_{1}=\left[\begin{array}{ccc} \cos\theta_{1}\cos\theta_{2} & -\sin\theta_{1} & \cos\theta_{1}\sin\theta_{2}\\ \sin\theta_{1}\cos\theta_{2} & \cos\theta_{1} & \cos\theta_{1}\sin\theta_{2}\\ -\sin\theta_{2} & 0 & \cos\theta_{2} \end{array}\right] \end{array}\right.$$

The bending deformation following the discretization under the assumed mode method is expressed as:

(6) $$v_{1}=a_{1}\sin\frac{\pi x_{1}}{l_{21}\cos\theta_{a}}+a_{2}\sin\frac{2\pi x_{1}}{l_{21}\cos\theta_{a}}$$

The kinetic energy of the thigh *l*_21_ segment is:

(7) $$T_{21}=\frac{1}{2}\int_{0}^{l_{21}\cos\theta_{a}}\rho {\dot{r}}_{1}{}^{T}{\dot{r}}_{1}dl=\frac{1}{2}{\dot{q}}_{2}{}^{T}M_{21}{\dot{q}}_{2}$$

Similarly, the kinetic energy *T*_22_ of the thigh *l*_22_ and the kinetic energy *T*_3_ of the shin can be obtained. The total kinetic energy of the rigid flexible coupling leg linkage system is

(8) $$T=T_{1}+T_{21}+T_{22}+T_{3}$$

where $T_{1}=\frac{1}{2}J_{0}{\dot{\theta }}_{1}^{2}$, $J_{0}$ is the rotational inertia of the base node. The generalized coordinates and their derivatives in the entire single leg rigid flexible coupling system are $q={\left[\begin{array}{ccccccc} \theta_{1} & \theta_{2} & \theta_{3} & a_{1} & a_{2} & b_{1} & b_{2} \end{array}\right]}^{T}\dot{q}={\left[\begin{array}{ccccccc} {\dot{\theta }}_{1} & {\dot{\theta }}_{2} & {\dot{\theta }}_{3} & {\dot{a}}_{1} & {\dot{a}}_{2} & {\dot{b}}_{1} & {\dot{b}}_{2} \end{array}\right]}^{T}$. The total mass matrix for a single leg is obtained by combining the mass matrices of the base, flexible thigh, and flexible calf according to their respective generalized coordinates, derived in Appendix A.

The elastic potential energy of the flexible thigh and flexible calf is as follows:

(9) $$V=\frac{1}{2}E_{1}I_{1}{\int_{0}^{l_{21}\cos\theta_{a}}\left[\frac{\partial^{2}u_{1}}{\partial x_{1}{}^{2}}\right]}^{2}dx_{1}+\frac{1}{2}E_{2}I_{2}{\int_{l_{21}\cos\theta_{a}}^{l_{2}}\left[\frac{\partial^{2}u_{2}}{\partial x_{2}{}^{2}}\right]}^{2}dx_{1}+\frac{1}{2}E_{1}I_{1}{\int_{0}^{l_{21}\cos\theta_{a}}\left[\frac{\partial^{2}u_{3}}{\partial x_{3}{}^{2}}\right]}^{2}dx_{1}$$

*E* is the elastic modulus of the leg material, where *E*_1_ *= E*_2_ *= E*_3_, and *I*_1_, *I*_2_, *I*_3_ are the average moment of inertia of each cross section. Equation (9) can be deformed by using generalized coordinates:

(10) $$V=\frac{1}{2}q^{T}Kq$$

where ***K*** is the stiffness matrix, derived in Appendix B.

The gravitational potential energy of the system can be expressed as:

(11) $$G=m_{1}g\frac{l_{1}}{2}+m_{2}g\frac{l_{2}}{2}\sin\theta_{2}+m_{3}g(l_{2}\sin\theta_{2}-\frac{l_{3}}{2}\cos(\theta_{2}-\theta_{3}))$$

The Lagrange equation is expressed as:

(12) $$\frac{d}{dt}(\frac{\partial L}{\partial \dot{q}})-\frac{\partial L}{\partial q}=Q$$

where ***L*** = ***T*** − ***G*** − ***V***, and ***Q*** represents all the generalized forces in the system.

It can be obtained from Equation (12):

(13) $$M\ddot{q}+\dot{M}\dot{q}-\frac{\partial }{\partial q}(\frac{1}{2}{\dot{q}}^{T}M\dot{q})+Kq=Q_{F}$$

The generalized driving force array ***Q_F_*** is derived from the principle of virtual work:

(14) $$Q_{F}=\left[\begin{array}{ccccccc} \tau_{1} & \tau_{2}-\tau_{3} & \tau_{3} & \tau_{2}\frac{\pi }{l_{21}\cos\theta_{a}}+\tau_{3}\frac{\pi }{l_{22}\cos\theta_{b}} & \tau_{2}\frac{\pi }{l_{21}\cos\theta_{a}}-\tau_{3}\frac{\pi }{l_{22}\cos\theta_{b}} & \tau_{3}\frac{\pi }{l_{3}} & \tau_{3}\frac{2\pi }{l_{3}} \end{array}\right]$$

## 3. Layered Control of Rigid–Flexible Coupling Characteristics

This section introduces a hierarchical control framework for robots with rigid–flexible coupling characteristics. It primarily includes a trajectory planning layer for a foot-end trajectory with flexible deformation feedforward compensation. To maintain the stability of the robot’s torso, there is a force planning layer that solves for the desired torque of each leg based on the torso’s center-of-mass trajectory and torso posture tracking. Finally, there is an execution layer of the controller, which includes a controller for precisely controlling the hydraulic drive unit, and an impedance controller that adapts to the external environment.

### 3.1. Feedforward Compensation

Due to the flexible deformation effect causing a change in the leg structure, the real-time position of the center of gravity is not easily computed. Therefore, this paper determines the position of the center of gravity during operation through a nonlinear neural-network model. The foot-end force of the supporting leg and the posture of the torso are used as inputs to the prediction model, and the output is the CoM trajectory. The extreme learning machine (ELM) algorithm effectively overcomes the local optima traps commonly found in gradient algorithms [34]. It is a special type of single-hidden-layer feedforward neural network (SLFN), with only one hidden layer similar to a neuronal layer.

The motion trajectory of the trunk is determined by the trajectory of the supporting foot, so the motion trajectory of the trunk can be determined based on the trajectory planning of the foot end. If the translation of the trunk is Δx and Δy, then we can obtain:

(15) $$F_{iz}=A^{-1}\cdot \left(\begin{array}{c} mg\\ 0\\ 0 \end{array}\right)+\Delta x^{\prime }\cdot {A^{\prime }}^{-1}\cdot \left(\begin{array}{c} 0\\ mg\\ 0 \end{array}\right)+\Delta y^{\prime }\cdot {A^{\prime }}^{-1}\cdot \left(\begin{array}{c} 0\\ 0\\ mg \end{array}\right)$$

where $A^{\prime }=\left(\begin{array}{ccc} 1 & \cdots & 1\\ x_{1} & \cdots & x_{n}\\ y_{1} & \cdots & y_{n} \end{array}\right)$, 3≤n≤6.

Equation (15) can be expressed as:

(16) $$F_{iz}=f_{co}+\Delta x^{\prime }\cdot f_{c1}+\Delta y^{\prime }\cdot f_{c2}$$

where $f_{co}$ is the vector of the vertical force at the foot at the initial moment of support; $f_{c1}$ and $f_{c2}$ are the normal vectors of the translation amount during the support process; $\Delta x^{\prime }$ and $\Delta y^{\prime }$ are the translation amounts of the trunk’s center of gravity relative to the foot-end point.

From Equation (16), in any support process, the vertical force exerted at the end of the foot is linearly related to the amount of translation of the torso. Therefore, the foot-end force can be predicted by the motion trajectory of the trunk, then the predicted value of the end-of-foot force can be derived from the Kalman filter [35], and the Kalman prediction equation is embodied in Appendix C.

Based on the predicted foot-end force, the deformation is described by the dynamics model of the leg and the intrinsic characteristic stiffness of the leg structure. The error of the foot-end-to-leg datum can be expressed as:

(17) $$E(T_{i})=\frac{\partial T_{i}}{\partial \theta_{1}}\cdot E(\theta_{1})+\frac{\partial T_{i}}{\partial \theta_{2}}\cdot E(\theta_{2})+\frac{\partial T_{i}}{\partial \theta_{3}}\cdot E(\theta_{3})$$

where *θ* is the angle between each component driven by the servo cylinder. The size of each oil cylinder of a single leg can be calculated based on its geometry, that is, the coordinate error of the landing point can be expressed by the size of the oil cylinder.

Therefore, the target trajectory of the robot after compensation is:

(18) $$x_{mc}=x_{m}+\Delta E_{f}$$

where *x_m_* is the desired trajectory of the foot end, and *x_mc_* is the actual trajectory entered by the controller.

### 3.2. Torque Control for Rigid–Flexible Coupled Robots

**Figure 3:**
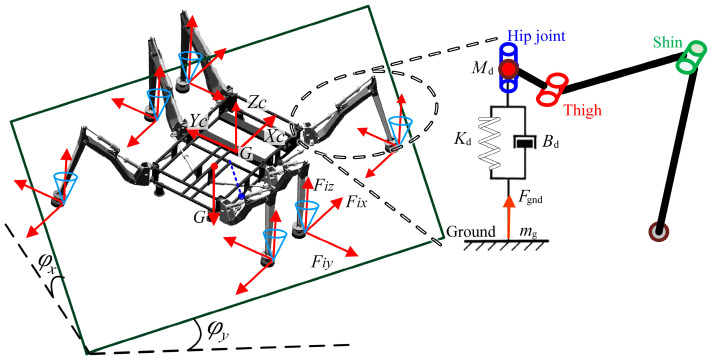
Force analysis of the robot and virtual impedance model of legs.

This paper proposed a multi-control model switching strategy, which includes precise force control of single-leg virtual impedance and hydraulic drive units. This strategy provided segmented control over robot movements. When the leg transitioned from the swing phase to the initial stage of the support phase, impedance control was employed to quickly track the foot-end force. When the foot-end force error reached a set threshold, it was assumed that the environment could provide sufficient support force for the system. At this point, the controller switched to the force controller of the hydraulic drive unit. The robot’s smooth movement was achieved through the coordinated control of the two. The impedance control abstracted the robot’s leg as a virtual mass-spring-damping model, as shown in Figure 3. Based on the real-time force ***F***, the impedance control model returned the correction amount of displacement *x*_1_, which was then added to the expected motion displacement *x*_2_ to generate a new displacement *x*_3_.

Given that the robot speed is relatively low and its motion state is smooth, the motion can be analyzed as quasi-static, as shown in Figure 3. On an inclined surface, the force equation for each foot end can be expressed as:

(19) AF=W

where $F={\left[\begin{array}{ccccc} F_{1x} & F_{1y} & F_{1z} & \ldots & F_{nz} \end{array}\right]}^{T}\in R^{3n}$ is the representation of the force at the foot end of each leg in the three coordinate directions; $W=[\begin{array}{cccccc} F_{x} & F_{y} & F_{z} & M_{x} & M_{y} & M_{z} \end{array}]$ is the force and the torque applied to the robot; $A=\left[\begin{array}{cccc} I_{3} & I_{3} & \ldots & I_{3}\\ {Ρ}_{1} & {Ρ}_{2} & \ldots & {Ρ}_{j} \end{array}\right]\in R^{6\times 3n}$, in which I is the unit matrix, and $P_{j}=[\begin{array}{ccc} {}^{G}x_{j3} & {}^{G}y_{j3} & {}^{G}z_{j3} \end{array}]$, where ^G^*x_j_*_3_, ^G^*y_j_*_3_, and ^G^*z_j_*_3_ represent the coordinate values of the foot end in each direction relative to the center of mass.

When a hexapod robot walks on real terrain, the foot-end forces in the support phase must satisfy the friction constraints to reduce slippage. Because the size of the foot end is negligible compared to the overall size of the hexapod robot, the contact between the foot end and the ground is assumed to be a point contact.

(20) $$\sqrt{F_{ix}^{2}+F_{iy}^{2}}\leq \xi F_{iz}^{2}$$

where ξ is the static friction coefficient.

Additionally, the support-phase foot-end force *F_iz_* must satisfy the following constraints:

(21) $$F_{iz}\geq 0$$

The elastic deformation of the flexible-leg structure is influenced by the reaction force at the robot’s foot end. Consequently, achieving a uniform distribution of force across each leg can reduce the elastic deformation of each supporting leg. Therefore, the square sum of the difference in force between each leg is used as the optimization target, as shown below:

(22) $$\left\{ \begin{array}{l} \min\;(F-\bar{G}{)}^{T}\;(F-\bar{G})\\ s.t.\;AF=W\\ \;EF\leq 0 \end{array}\right.$$

where $\bar{G}=G/n$, *n* represents the number of support legs of the robot, and *G* is the gravitational force acting on the robot.

The simplified impedance control model of the robot is shown in Figure 3, and the mathematical expression of the impedance control model is:

(23) $$F_{r}-F=M_{d}(\ddot{X}-{\ddot{X}}_{r})+B_{d}(\dot{X}-{\dot{X}}_{r})+K_{d}(X-X_{r})$$

where ***M****_d_*, ***B****_d_*, and ***K****_d_* are the target inertia, target damping, and target stiffness matrices of the impedance control model, ***F*** and ***F****_r_* represent the actual and desired contact forces, respectively, and ***X*** and ***X****_r_* represent the actual and desired positions, respectively.

The transfer function of Equation (23) is:

(24) $$G(s)=\frac{X(s)}{F(s)}=\frac{1}{Ms^{2}+Bs+K}$$

During the robot’s movement, when there is a protrusion on the ground, the foot makes contact with the ground earlier, and the foot pressure sensor provides a certain amount of force feedback. At this point, the anticipated force on the foot is zero, and following impedance control, the foot end retracts to some extent. When there is a depression on the ground, a delay in contact exists between the foot end and the ground. After impedance control, the foot end extends to compensate for the tilt of the robot’s body. Although impedance control can enhance the robot’s terrain adaptability, its effectiveness depends on the parameter settings. For legged robots with rigid–flexible coupling characteristics, relying solely on impedance control will inevitably cause oscillation in the robot’s torso when the robot’s leg undergoes flexible deformation.

In order to reduce the robot vibrations caused by the coupling of the flexible-leg deformation and the robot’s trunk, a force control model mapping from task space to joint space is established. Joint acceleration, torque, and foot–ground interaction force are treated as decision variables, and the desired output torque of the strongly coupled dynamic system is solved using the quadratic programming (QP) optimization algorithm. The robot’s trunk can be considered a rigid body, and the momentum of each rod is mapped into the centroid space with the centroid as the origin, leading to the derivation of the centroid momentum model.

In this paper, the spatial notation of six-dimensional vectors is used to represent the angular and linear momentum of the rigid body uniformly as:

(25) $$h_{i}=\left[\begin{array}{c} h_{i}^{angle}\\ h_{i}^{line} \end{array}\right]=\left[\begin{array}{cc} {\bar{I}}_{i} & m_{i}S(r_{i})\\ m_{i}S{\left(r_{i}\right)}^{T} & m_{i}E_{3} \end{array}\right]\left[\begin{array}{c} w_{i}\\ v_{i} \end{array}\right]={\hat{I}}_{i}{\hat{v}}_{i}$$

where $h_{i}$ is the rigid-body momentum, $S(r_{i})$ is the skew-symmetric cross product matrix, ***E_3_*** is the unit diagonal matrix, ${\bar{I}}_{i}$ is the spatial inertia, ${\hat{I}}_{i}$ is the inertia tensor, ${\hat{v}}_{i}$ is the velocity of the motion at the origin *o_i_* of the coordinate system, $w_{i}$ is the angular velocity vector, $h_{i}^{angle}$ is the linear momentum, and $h_{i}^{line}$ is the angular momentum.

The total momentum of the robot’s multi-rigid-body system is the sum of the momenta of all rod elements projected onto the centroid coordinates, which can be expressed as:

(26) hG=∑i=1NXiGThi=∑i=1NXiGTI^iJ^iq˙=AGq˙

where $h_{G}$ is the momentum space transformation matrix from coordinate system o_*i*_ to centroid coordinate system; $A_{G}$ is the centroidal momentum matrix.

The kinetic equations for the CoM were obtained by taking a first-order derivative of Equation (26):

(27) $$f_{G}={\dot{h}}_{G}=A_{G}\ddot{q}+{\dot{A}}_{G}\dot{q}$$

where $f_{G}$ is the vector of the net external force at the centroid.

Hexapod robots belong to the floating-base system, and its dynamic equations are expressed as follows:

(28) $$M(q)\ddot{q}+C(q,\dot{q})\dot{q}+G(q)=S^{T}\tau_{t}+J_{\lambda }^{T}\lambda$$

where λ is the constraint forces due to contact.

The gravity term and the force of the legs on the body are equivalent to the net external force of the floating body, and the dynamic equation of the floating body can be simplified as:

(29) $${P^{\prime }}_{1}(M\ddot{q}+C\dot{q})=\tau_{t}=\phi_{1}^{T}f_{t}$$

where $P_{1}=[\begin{array}{cc} E_{6} & 0_{6\times n} \end{array}]$, $\phi_{1}$ is the relationship torque between the net external force and the generalized force acting on the floating body, $\tau_{t}$ is the generalized force torque acting on the floating body, and $f_{t}$ is the net external force acting on the floating body.

Since the centroid coordinate axis is parallel to the inertial system coordinate axis, the system’s net external force acting at the centroid can be directly described using the task space system net external force:

(30) $$f_{G}^{*}=\left[\begin{array}{c} {\dot{h}}_{G}^{angle}\\ {\dot{h}}_{G}^{line} \end{array}\right]=\left[\begin{array}{c} {\dot{h}}_{G}^{angle*}\\ m_{G}{\ddot{c}}^{*} \end{array}\right]$$

Due to the flexible deformation of the legs and the movement of the robot, there is a deviation between the actual trajectory and the ideal centroid trajectory, so the centroid trajectory PD feedback compensation is introduced as follows:

(31) $$f_{G}^{*}=\left[\begin{array}{c} {\dot{h}}_{G}^{angle}+k_{p,\theta }(h_{G}^{angle}-h_{G}^{{angle}^{\prime }})+k_{d,\theta }({\dot{h}}_{G}^{angle}-{\dot{h}}_{G}^{{angle}^{\prime }})\\ m_{G}[{\ddot{c}}^{*}k_{p,c}(c-c^{\prime })+k_{d,c}(\dot{c}-{\dot{c}}^{\prime })] \end{array}\right]$$

When calculating inverse dynamics, the position tracking error needs to be considered to ensure that the torque calculation in the joint drive space controls the task space body pose. The relationship between the task space body pose and the joint space generalized acceleration can be expressed as:

(32) $${\ddot{x}}_{b}^{*}=J_{b}\ddot{q}+{\dot{J}}_{b}\dot{q}$$

where *x_b_* is the acceleration vector of the expected body pose, and $J_{b}$ is the Jacobian matrix from joint space to body pose.

In this paper, the PD feedback control model is applied to the positional trajectory tracking of the torso. The robot’s motion process is affected by flexible deformation, position control error, and the nonlinear strong coupling between foot–ground interaction, so this paper uses a quadratic programming optimization algorithm to solve the problem. Its optimization paradigm is expressed as follows:

(33) $$\left\{ \begin{array}{l} \underset{\gamma }{\min}\frac{1}{2}\gamma^{T}Q\gamma +r^{T}\gamma \\ s.t.\;CE\gamma +ce=0\\ CI\gamma +ci\geq 0 \end{array}\right.$$

The objective functions are all expressed in the form of least squares optimization, and the Hessian matrix and gradient term of the objective function are $Q=A^{T}A$, $R=-A^{T}H$, where $A=\left[\begin{array}{c} w_{0}A_{G}\\ w_{1}J_{b} \end{array}\right]$, and $R=\left[\begin{array}{c} \eta_{0}[f_{G}^{*}-{\dot{A}}_{G}\dot{q}]\\ \eta_{1}[f{\ddot{x}}_{b}+k_{p,b}(x_{b}-x_{b}{}^{\prime })+k_{d,b}({\dot{x}}_{b}-{\dot{x}}_{b}{}^{\prime })-{\dot{J}}_{b}\dot{q}] \end{array}\right]$

The decision variables are:

(34) $$\gamma =[\begin{array}{cc} \ddot{q} & \tau_{t} \end{array}]$$

The inequality constraints of the optimization algorithm include generalized acceleration constraints and joint torque constraints, while the equality constraint is the centroid dynamics equation constraint. The system’s optimal torque *τ_t_* can be obtained through a numerical solution algorithm. Then, in combination with the dynamic model of the flexible-leg mechanism, the desired input torque *τ_s_* for the hydraulic drive unit is obtained.

The desired output force of the cylinder is:

(35) $$F_{c}=J_{0}{}^{-1}\tau_{s}$$

where $J_{0}{}^{-1}$ is the Jacobi matrix of the cylinder force reaction joint moment of the leg.

According to the force analysis of the hydraulic drive unit in Appendix D, the state variables of the system are selected as $x=[x_{1},x_{2},x_{3},x_{4}]=[x_{p},{\dot{x}}_{p},p_{1},p_{2}]$. The state space equations are as follows:

(36) $$\left\{ \begin{array}{l} {\dot{x}}_{1}=x_{2}\\ {\dot{x}}_{2}=(-K_{L}x_{1}-B_{t}x_{2}+A_{1}x_{3}-A_{2}x_{4}-F_{c})/m_{t}\\ {\dot{x}}_{3}=\Gamma_{1}u+\frac{\beta_{e}}{V_{1}}(-A_{1}x_{2}-(C_{ip}+C_{ep})x_{3}+C_{ip}x_{4}+d)\\ {\dot{x}}_{4}=\Gamma_{2}u+\frac{\beta_{e}}{V_{2}}(-A_{2}x_{2}+C_{ip}x_{3}-(C_{ip}+C_{ep})x_{4}) \end{array}\right.$$

where

$$\Gamma_{1}=\frac{K_{sv}\beta_{e}C_{d}w}{V_{1}}\sqrt{\frac{2}{\rho }\left|\frac{(1+\mathrm{sgn}(x_{v}))p_{s}}{2}+\frac{(-1+\mathrm{sgn}(x_{v}))p_{0}}{2}-\mathrm{sgn}(x_{v})x_{3}\right|}$$

$$\Gamma_{2}=-\frac{K_{sv}\beta_{e}C_{d}w}{V_{2}}\sqrt{\frac{2}{\rho }\left|\frac{(1-\mathrm{sgn}(x_{v}))p_{s}}{2}+\frac{(-1-\mathrm{sgn}(x_{v}))p_{0}}{2}+\mathrm{sgn}(x_{v})x_{4}\right|}$$

The equation of state of the system can be expressed as:

(37) $$\{\begin{array}{c} \dot{x}=Ax+Bu\\ y=Cx \end{array}$$

The synovial surface and control rate were set to the following form:

(38) $$e={\hat{F}}_{q}-F_{q}$$

The sliding mode surface is defined as follows:

(39) s=e

Let $\dot{s}=0$, the equivalent control *v* of the system is:

(40) $$\dot{s}=\dot{e}={\dot{\hat{F}}}_{q}-{\dot{F}}_{q}$$

The switching function of the sliding control is:

(41) $$v_{s}=-\varepsilon \mathrm{sgn}(s)-ks$$

The function is established as follows using the theory of Lyapunov functions:

(42) $$\begin{array}{l} V=\frac{1}{2}s^{2},s=e\\ \dot{V}=s\dot{s}=s\dot{e}=s(-\varepsilon \mathrm{sgn}(s)-ks)=-\varepsilon \left|s\right|-ks^{2}\leq 0 \end{array}$$

The established slip film control system that can be obtained from Equation (42) is stable. However, the sign function in the sliding film control will cause vibration and chattering to the system, so the following boundary layer function is chosen to replace the sign function:

(43) $$sat(\frac{s}{\mu })=\{\begin{array}{c} \begin{array}{cc} \mathrm{sgn}(s/\mu ) & \left|s/\mu \right|\geq 1 \end{array}\\ \begin{array}{cc} s/\mu & \left|s/\mu \right|<1 \end{array} \end{array}$$

where μ is the boundary layer function.

The control rate of the synovial system is:

(44) $$u=b^{-1}[{\dot{\hat{F}}}_{q}-\varepsilon sat(s/\dot{\mu })-ks-Ax]$$

## 4. Results and Discussion

### 4.1. Simulation

The heavy-duty hexapod robot is loaded with a total weight of 3500 kg. The mass distribution of the torso is simulated by adjusting the corresponding mass blocks in Adams according to the actual center-of-gravity position, and the fine flexible body models of the thighs and calves are created by using the finite-element software and imported into Adams through the Modal Neutral File (MNF). A control algorithm model of the system was constructed in Matlab/Simulink, and a hydraulic system model based on a jet pipe feedback two-stage servo valve control cylinder was also constructed. The force and displacement output by the cylinder were used as driving signals for the robot in the Adams environment.

**Figure 4:**
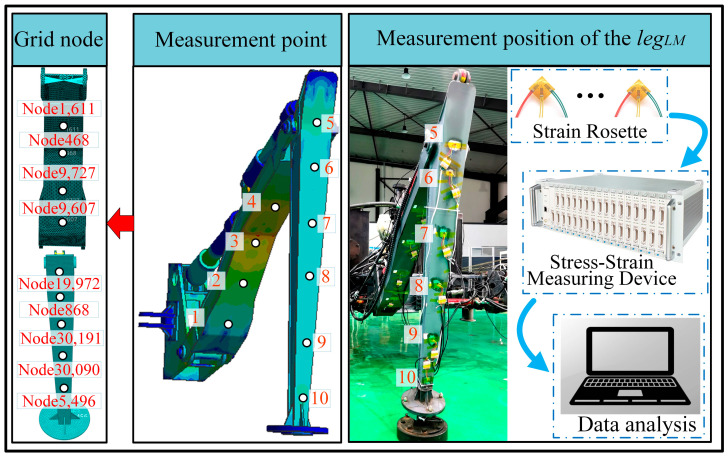
Experiments on stress testing of the leg.

A dynamic strain gauge and three-axis right-angle strain flower were used to test the dynamic stress and strain of the legs of a hexapod robot. As shown in Figure 4, the testing scheme design for dynamic stress and strain in this article is presented. According to the distribution characteristics of the stress cloud map analyzed by simulation, the experimental area is divided, and the number and position of the measured points are determined. Due to the maximum load on the middle leg during two-step walking, the left middle leg is selected as the experimental leg, and a total of 10 strain patterns are pasted on the thigh and calf. The measurement point positions are determined in the experiment in Hypermesh and described with node information, making it easy to find the corresponding areas in Adams during post-processing comparative analysis.

**Figure 5:**
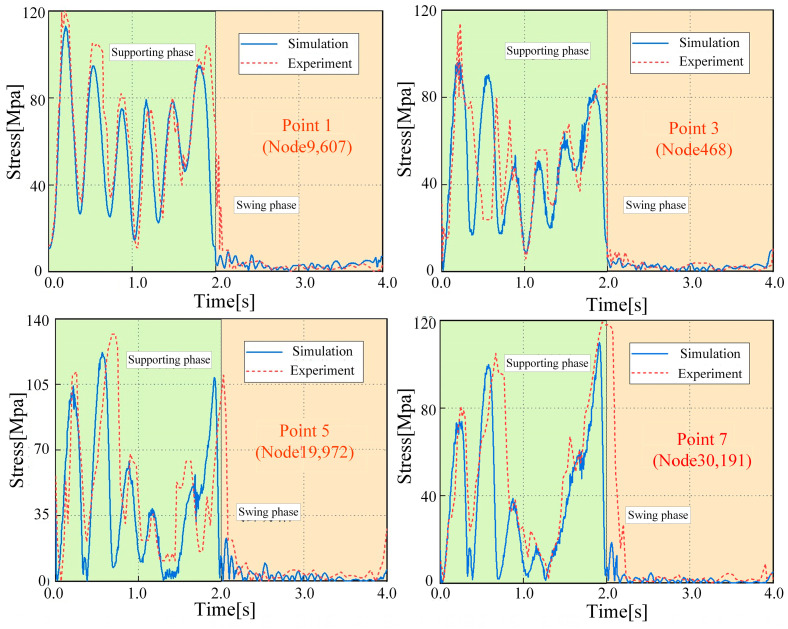
Results of stress tests on LM leg.

In the experiment, strain signals from different directions were collected and processed into equivalent stress for each measurement point and then compared with the simulation results. The comparison curves for some of the measurement points are shown in Figure 5. It can be observed that, although there are certain errors and fluctuations between the experimental curve and the simulation curve, these are due to influences from factors such as the on-site environment, foot contact stiffness, joint friction coefficients, wire fixing methods, and patch operations. The overall trend remains highly consistent, thereby validating the accuracy of the rigid–flexible coupling simulation model and the rationality of subsequent analysis based on this model.

**Figure 6:**
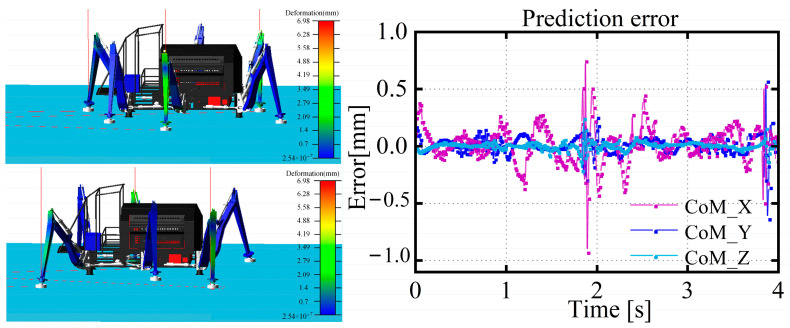
Simulation scenarios and predictive results of the neural-network model.

A two-step forward mode was adopted as the robot’s motion planning during the simulation process, with three legs serving as the supporting legs and the other three as the swinging legs. The gait cycle was set to 2 s, with a duty factor of 0.5, a stride length of 1 m, and the foot end of the swinging leg was elevated by 0.2 m in the z direction. The robot’s foot trajectory utilized a low-impact foot trajectory method, constrained by the initial position and speed, and the acceleration adopts an even sine function. As shown in Figure 6, the prediction results of the centroid prediction algorithm proposed in this paper are displayed. It can be seen that the real-time error of the CoM in three directions does not exceed 1 mm, which can be applied to the feedforward compensation control of flexible deformation.

**Figure 7:**
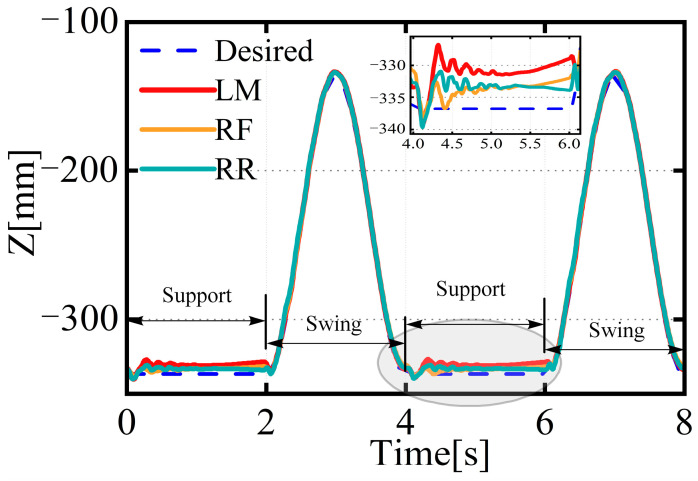
Actual trajectory of the foot end of the supporting leg with respect to the origin of the coordinate system of the supporting leg.

Figure 7 shows the actual z-direction positions of the foot end of the three supporting legs relative to the leg base. It can be seen that there is a deviation between the actual positions of the foot ends of the three legs in the support state and the positions calculated through position sensor feedback and the kinematic model. A strong coupling relationship exists between the supporting legs and the rigid body. If the controller plans according to the foot-end trajectory that has not undergone deformation, it will lead to the internal force coupling of the leg itself and the internal force coupling of all supporting legs and the body, causing instability in the entire body. It can be observed that the flexible deformation of the three legs changes with the body’s movement, with the LM leg experiencing the most significant flexible deformation, due to the fact that the force exerted on this leg is the greatest among all supporting legs.

**Figure 8:**
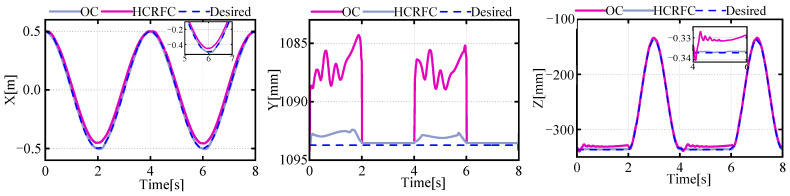
Curves of the desired and actual trajectories of the foot.

The feedforward compensation control proposed in this paper was validated through simulation, with the results of the left middle (LM) leg shown in Figure 8. As original controllers (OCs) could only improve the control accuracy of the drive unit and were powerless against the flexible deformation of the leg mechanism, there was a corresponding deviation in the foot trajectory relative to the base position from the expected trajectory. However, the position information of the feedback controller could not reflect this deviation, thereby exacerbating the coupling between the leg and the body, leading to vibrations throughout the system. The method of feedforward compensation for flexible deformation proposed in this paper significantly improved the tracking accuracy of the robot’s foot trajectory relative to the leg base. It reduced the real error of the foot trajectory relative to the leg base.

**Figure 9:**
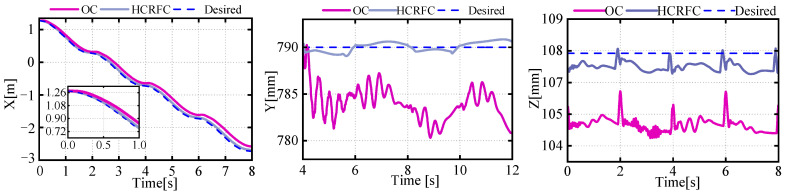
Curves of the desired and actual trajectories of the CoM.

Figure 9 illustrates the changes in the position of the centroid during the robot’s motion. It can be seen that the robot’s flexible-leg deformation would cause a deviation in the CoM. The error in the z direction was present when the gait transitioned to a state with multiple legs providing support simultaneously, resulting in a temporary reduction in the body’s z-direction deviation, which then returned to the normal error level. Meanwhile, corresponding deviations also existed in the trajectories of the CoM in the x and y direction, which was due to the different deformation states of the supporting legs exerting different forces on the robot’s body, causing a shift in the movement trajectory of the CoM. The HCRFC controller was capable of controlling the CoM more smoothly and reducing the deviation of the CoM during the robot’s movement.

**Figure 10:**
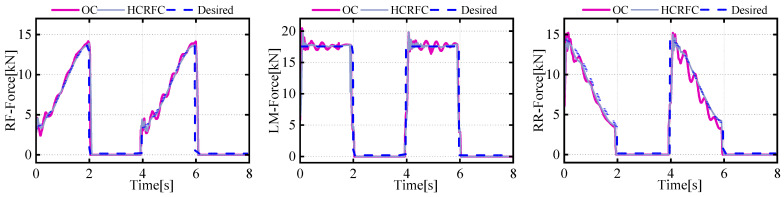
Curve of tracking effect of desired and actual force.

As shown in Figure 10, a force deviation occurred during the robot’s gait transition and movement. The LM leg could be seen to bear the highest force, and the force on the RR and RF legs on one side changed with the CoM, but the overall vibration of the robot caused force fluctuations at the foot end. Due to the flexible deformation of the leg, a deviation exists between the actual position and the feedback position, which could easily lead to force oscillations during impedance control and impede the tracking of the desired force. It can be observed that after adopting the method of HCRFC mentioned in Section 3.2, although certain force deviations may occur during the gait transition process, these deviations can be quickly reduced to achieve a stable state, facilitating smooth movement of the robot.

Based on the analysis mentioned above, it is understood that when the robot body experiences vibrations, the trajectory of the center of mass will also deviate. This deviation concurrently affects the force exerted on the legs. Thus, the trajectory of the center of mass can effectively reflect the system’s motion state. In order to show the performance of the proposed control method more clearly, this study simulated different scenarios for analysis, using the maximum deviations in the three directions of the CoM as the targets. The results, shown in Table 2, indicated that the control strategy proposed in this paper effectively improved the performance of robots with rigid–flexible coupling characteristics.

### 4.2. Experiments

**Figure 11:**
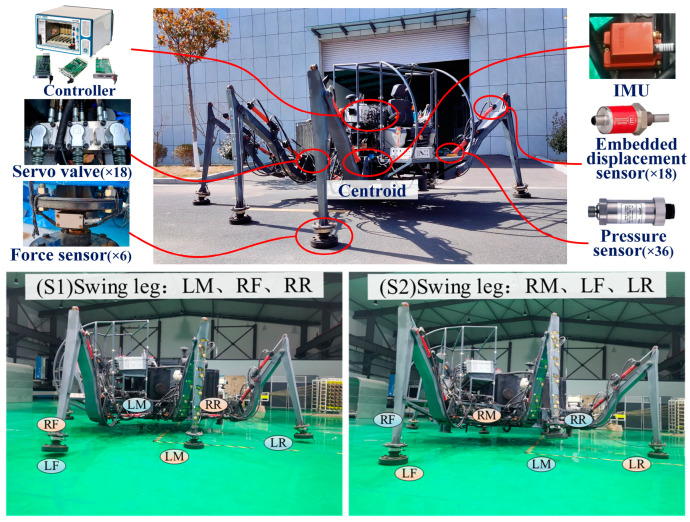
Diagrams of experimental platforms and scenarios.

The control strategy proposed in this paper was validated on a real hexapod robot, as shown in Figure 11. The control system was built in a hardware-in-the-loop configuration, where the control algorithm model was established on a host computer through MATLAB/Simulink and then deployed to an embedded industrial computer. The embedded industrial control host used the VxWorks system, was equipped with corresponding PCI cards, and had ample development resources, enabling real-time data exchange with multiple distributed systems. The parameters of the experimental motion trajectory were the same as the simulation conditions, with a bipedal walking pattern being used. The gait cycle was 2 s, the stride was 1m, and the leg lift height was 0.2 m. Due to the noise in the sensor data, a Butterworth filter was used for smoothing, with a delay of 0.2 ms.

**Figure 12:**
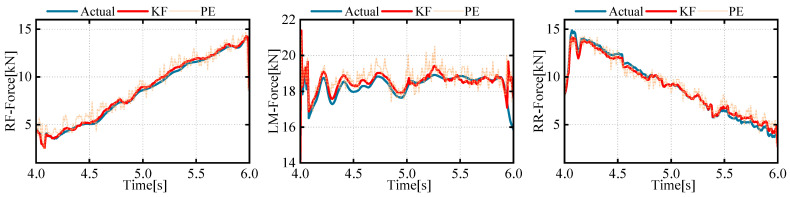
Curves of predicted and actual end-foot forces.

This paper analyzes the expected foot forces that provide a direct reference for feedforward compensation, as the actual position of the foot and leg data in a realistic robot is difficult to measure. Figure 12 shows the estimation results of future foot force based on the centroid trajectory and current foot force using the Kalman prediction algorithm. It can be seen that the centroid trajectory based on foot force and estimation proposed in this article can accurately predict the foot force at the next moment.

**Figure 13:**
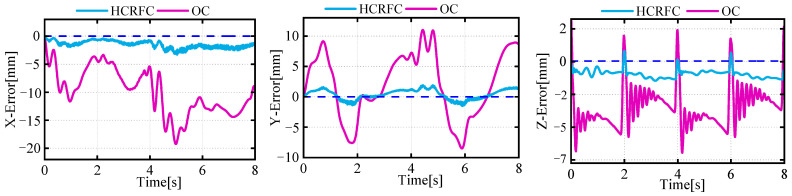
Curves of motion deviation of the CoM.

**Figure 14:**
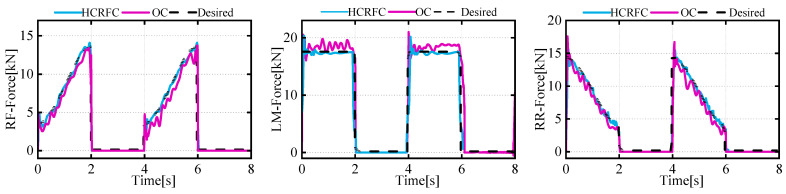
Curve of tracking effect of desired and actual force.

Figure 13 and Figure 14 present the data on the CoM position and foot-end force in the experiment. The trajectory of the CoM was predicted using a neural-network model, while the data on foot-end force were directly measured by sensors. It was observed that, in the initial stages, the robot still experienced transient vibrations due to the impact system, causing certain deviations in all three directions and corresponding fluctuations in the foot-end force of the supporting legs. After employing the HCRFC control, the robot demonstrated improved trajectory tracking performance in all three directions of the CoM, and the foot-end force of each supporting leg also reached a stable state more quickly. However, compared to the simulation results, its control performance declined somewhat, because the robot had to consider disturbances such as friction, processing errors, and other external environmental factors. These issues will be the focus of our future research. In summary, the experiment and simulation validated the performance of the HCRGC controller.

## 5. Conclusions

This paper proposed an HCRFC strategy for legged robots. Firstly, to reduce the deformation of the body and the flexible-leg end, a method of flexible deformation feedforward compensation is proposed. Then, to minimize the impact of leg deformation on the robot’s body, a dynamic component compensation control is designed that takes into consideration the centroid linear momentum, centroid angular momentum, and body trajectory. The CLM, CAM, and body trajectory are used as objective functions to solve for the desired driving torque through a quadratic programming optimization algorithm. Finally, a sliding mode controller is designed to achieve torque tracking of the hydraulic drive unit. This control strategy has been tested and simulated on a hexapod robot with a total weight of 3.5 tons. The results show that the method proposed in this paper can effectively reduce system vibrations. The control framework proposed in this paper can be widely applied in the field of legged robots.

In the future, we will focus on researching the decoupling control between each leg and joint in the support state. In addition, introducing energy-based posture adjustment into the existing framework is one of our upcoming goals.

## Figures and Tables

**Table 1:** D-H parameter of the leg.

Leg Section	*L_i_*	*d_i_*	*α_i_*	*θ_i_*
Hip joint	1.5 m	0	π/2	*θ*_1_
Thigh	1.5 m	0	0	*θ*_2_
Shin	0.33 m	0	0	*θ*_3_

**Table 2:** Table of HCRFC controller control performance.

	Level Road		Roll Angle = 10°		Pitch Angle = 10°	
	OC	LCRFC	OC	LCRFC	OC	LCRFC
Gx_Error (mm)	38.3	6.6	41.6	6.9	53.6	8.3
Gy_Error (mm)	11.6	1.4	14.3	1.6	12.3	1.4
Gz_Error (mm)	4.8	0.9	5.1	0.9	6.7	1.2

## Data Availability

No new data were created or analyzed in this study. Data sharing is not applicable to this article.

## Appendix A

The total mass matrix of the leg is represented as follows:

$$M=\left[\begin{array}{ccccccc} m_{11} & 0 & 0 & 0 & 0 & 0 & 0\\ 0 & m_{22} & m_{23} & m_{24} & m_{25} & m_{26} & 0\\ 0 & m_{32} & m_{33} & 0 & 0 & m_{36} & m_{37}\\ 0 & m_{42} & 0 & m_{44} & 0 & 0 & 0\\ 0 & m_{52} & 0 & 0 & m_{55} & 0 & 0\\ 0 & m_{62} & m_{63} & 0 & 0 & m_{66} & 0\\ 0 & 0 & m_{73} & 0 & 0 & 0 & m_{77} \end{array}\right]$$

where

$$\begin{array}{l} m_{11}={\tilde{m}}_{21}(\frac{a_{1}^{2}+a_{2}^{2}}{2}\sin^{2}\theta_{2}-\frac{l_{21}^{2}}{3}\cos\theta_{a}(\sin^{2}\theta_{2}-1)+\frac{l_{21}}{\pi }\cos\theta_{a}\sin2\theta_{2}(a_{1}-\frac{a_{2}}{2}))+{\tilde{m}}_{22}(\frac{a_{1}^{2}+a_{2}^{2}}{2}\sin^{2}\theta_{2}-\frac{l_{22}^{2}}{3}\cos\theta_{b}(\sin^{2}\theta_{2}-1)\\ +\frac{l_{22}}{\pi }\cos\theta_{b}\sin2\theta_{2}(a_{1}-\frac{a_{2}}{2}))+m_{3}(\frac{l_{21}^{2}\cos^{2}\theta_{a}+l_{22}^{2}\cos^{2}\theta_{b}}{2}\cos\theta_{2}^{2}-\frac{l_{3}}{3}(\sin^{2}\theta_{3}-1)+\frac{b_{1}^{2}+b_{2}^{2}}{2}\sin^{2}\theta_{3}+\frac{l_{3}}{\pi }\sin2\theta_{3}(b_{1}-\frac{b_{2}}{2})\\ +l_{2}\cos\theta_{2}(l_{3}\cos\theta_{3}+\frac{4b_{1}}{\pi }\sin\theta_{3}))+l_{1}^{2}(m_{2}\cos\theta_{2}+m_{3}\cos\theta_{3})+\frac{4l_{1}}{\pi }(m_{2}a_{1}\sin\theta_{2}+m_{3}b_{1}\sin\theta_{3})+J_{0};\\ m_{22}=\frac{m_{2}}{3}(l_{21}^{2}+l_{22}^{2}+\cos^{2}\theta_{a}+\cos^{2}\theta_{b}+\frac{a_{1}^{2}+a_{2}^{2}}{2})+m_{3}l_{2}^{2};m_{23}=m_{32}=m_{p}(l_{3}^{2}-l_{2}l_{3}\sin\theta_{3})-\frac{m_{3}l_{2}l_{3}}{2}\cos(\theta_{2}+\theta_{3})-\frac{2b_{1}}{\pi }l_{2}\sin(\theta_{2}+\theta_{3});\\ m_{24}=m_{42}=-\frac{{\tilde{m}}_{21}l_{21}\cos\theta_{a}+{\tilde{m}}_{22}l_{22}\cos\theta_{b}}{\pi };m_{25}=m_{52}=\frac{{\tilde{m}}_{21}l_{21}\cos\theta_{a}+{\tilde{m}}_{22}l_{22}\cos\theta_{b}}{2\pi };m_{26}=m_{62}=\frac{2m_{3}l_{2}\cos(\theta_{2}+\theta_{3})}{\pi };\\ m_{33}=m_{p}l_{3}{}^{2}+m_{3}(\frac{l_{2}^{2}}{3}+\frac{b_{1}^{2}+b_{2}^{2}}{2});m_{36}=m_{63}=\frac{m_{3}l_{3}}{\pi };m_{37}=m_{73}=\frac{m_{3}l_{3}}{2\pi };m_{44}=m_{55}=\frac{m_{2}}{2};m_{66}=m_{77}=\frac{m_{3}}{2} \end{array}$$

## Appendix B

The stiffness matrix of the leg is represented as:

$$K=\left[\begin{array}{ccccccc} 0 & & & & & & \\ & 0 & & & & & \\ & & 0 & & & & \\ & & & k_{44} & & & \\ & & & & k_{55} & & \\ & & & & & \frac{\pi^{4}}{2l_{3}^{3}}E_{3}I_{3} & \\ & & & & & & \frac{8\pi^{4}}{l_{3}^{3}}E_{3}I_{3} \end{array}\right]$$

where

$$k_{44}=\frac{\pi^{4}E_{1}I_{1}}{2{\left(l_{21}\cos\theta_{a}\right)}^{3}}+\frac{\pi^{4}E_{2}I_{2}}{2{\left(l_{21}\cos\theta_{a}+l_{22}\cos\theta_{b}\right)}^{3}}-\frac{\pi^{4}E_{2}I_{2}}{2{\left(l_{21}\cos\theta_{a}\right)}^{3}};k_{55}=\frac{8\pi^{4}E_{1}I_{1}}{{\left(l_{21}\cos\theta_{a}\right)}^{3}}+\frac{8\pi^{4}E_{2}I_{2}}{{\left(l_{21}\cos\theta_{a}+l_{22}\cos\theta_{b}\right)}^{3}}-\frac{8\pi^{4}E_{2}I_{2}}{{\left(l_{21}\cos\theta_{a}\right)}^{3}}$$

## Appendix C

The expression for the Kalman prediction algorithm is as follows:

(A1) $$\{\begin{array}{c} y_{t}=f_{iz}(y_{t-1})+w(t)\\ z_{t}=y_{t}+v_{t} \end{array}$$

where *w(t)* is the system state error and *v(t)* is the measurement error, and the model’s prediction step equation is:

(A2) $$\{\begin{array}{c} {{\hat{y}}^{\prime }}_{t}=f_{iz}({\hat{y}}_{t-1})\\ {{\hat{P}}^{\prime }}_{t}=N{\hat{P}}_{t-1}N^{T}+{\hat{Q}}_{t} \end{array}$$

where ${\hat{P}}^{\prime }$ is the covariance matrix of the current predicted state, ${\hat{Q}}_{t}$ is the learnable parameter in the model, and *N* is the Jacobi matrix of $f_{iz}(\cdot )$ with respect to $y_{t}$.

The equation for the updating step of the model is

(A3) $$\{\begin{array}{c} K_{t}={{\hat{P}}^{\prime }}_{t}{\left({{\hat{P}}^{\prime }}_{t}+{\hat{R}}_{t}\right)}^{-1}\\ {\hat{y}}_{t}={{\hat{y}}^{\prime }}_{t}+K_{t}({\hat{z}}_{t}-{\hat{y}}^{\prime })\\ {\hat{P}}_{t}=(I-K_{t}){{\hat{P}}^{\prime }}_{t} \end{array}$$

where ${\hat{R}}_{t}$ is the learnable parameter in the model, ${\hat{z}}_{t}$ is the observation of the target at moment *t*, and $K_{t}$ is the Kalman gain.

## Appendix D

An electro-hydraulic servo valve-controlled cylinder system was used as the hydraulic drive unit of the robot, and the flow equation of the servo valve is:

(A4) $$Q_{L}=K_{sv}x_{v}-K_{c}P_{L}$$

where *K_sv_* is the zero position flow gain of the directional valve, and *K_c_* is the pressure-flow coefficient of the directional valve at steady state.

The flow rate in the working chamber of the hydraulic cylinder is:

(A5) $$Q_{L}=A_{c}\frac{dy_{g}}{dt}+C_{L}p_{L}+\frac{V_{e}}{4\beta_{e}}\cdot \frac{dp_{L}}{dt}$$

In this paper, the force balance equations of the hydraulic cylinder piston and load are obtained by neglecting the nonlinear loads such as Coulomb friction:

(A6) $$A_{A}P_{A}-A_{B}P_{B}=M_{t}\frac{d^{2}y_{g}}{dt^{2}}+B_{t}\frac{dy_{g}}{dt}+K_{L}y_{g}+F_{L}$$

*M_t_* is the total mass of the hydraulic cylinder piston and load, b is the viscous damping coefficient of the load, *K_L_* is the spring stiffness of the oil, and *F_L_* is the external load force acting on the piston.
